# Supplementary material for: Effects of Pre-Treatment Using Plasma on the Antibacterial Activity of Mushroom Surfaces
Source: Foods. 2021 Aug 15;10(8):1888. doi: 10.3390/foods10081888 (PMC8394274; doi:10.3390/foods10081888)
Supplement: Supplementary file 1 [file foods-10-01888-s001.zip › 20210617 Mitra et al Supplementary R2.pdf]

Supplementary Information

Effects of pre-treatment using plasma on the antibacterial activity of mushroom surfaces

Sarmistha Mitra [1], Mayura Veerana [1], Eun-Ha Choi [1,2] and Gyungsoon Park [1,2,*]

[1] Plasma Bioscience Research Center and Department of Plasma-Bio and Display, Kwangwoon University, Seoul, 01897, Korea; sarmisthacu@gmail.com (S.M.); mayuraveerana@gmail.com (M.V.)

[2] Department of Electrical and Biological Physics, Kwangwoon University, Seoul, 01897, Korea; ehchoi@kw.ac.kr (E.C.); gyungp@kw.ac.kr (G.P.)

* Correspondence: gyungp@kw.ac.kr; Tel.: +82-2-940-8324

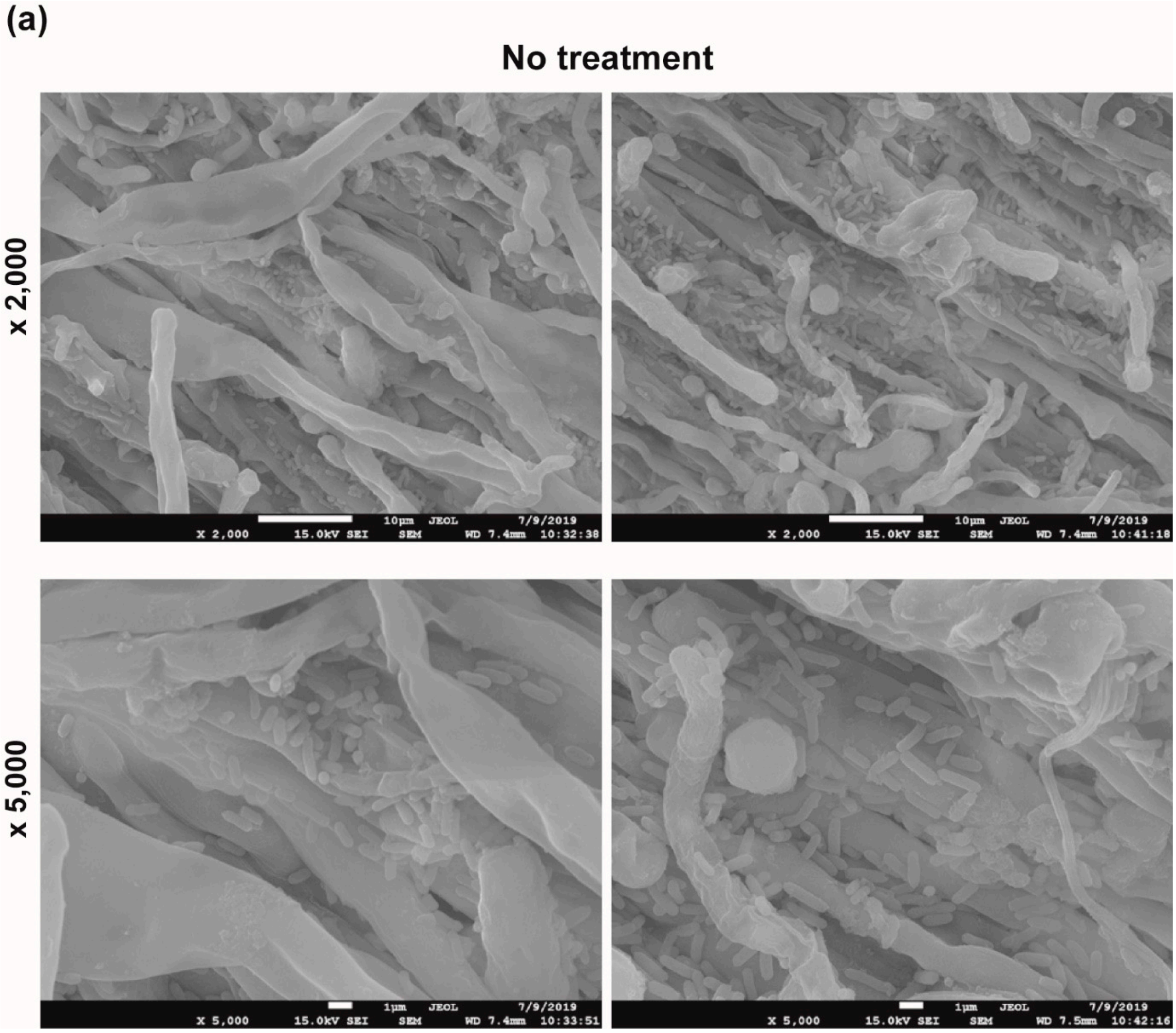
(a)
No treatment
x 2,000
x 5,000
10μm JEOL 7/9/2019
X 2,000 15.0kV SEI SEM WD 7.4mm 10:32:38
10μm JEOL 7/9/2019
X 2,000 15.0kV SEI SEM WD 7.4mm 10:41:18
1μm JEOL 7/9/2019
X 5,000 15.0kV SEI SEM WD 7.4mm 10:33:51
1μm JEOL 7/9/2019
X 5,000 15.0kV SEI SEM WD 7.5mm 10:42:16

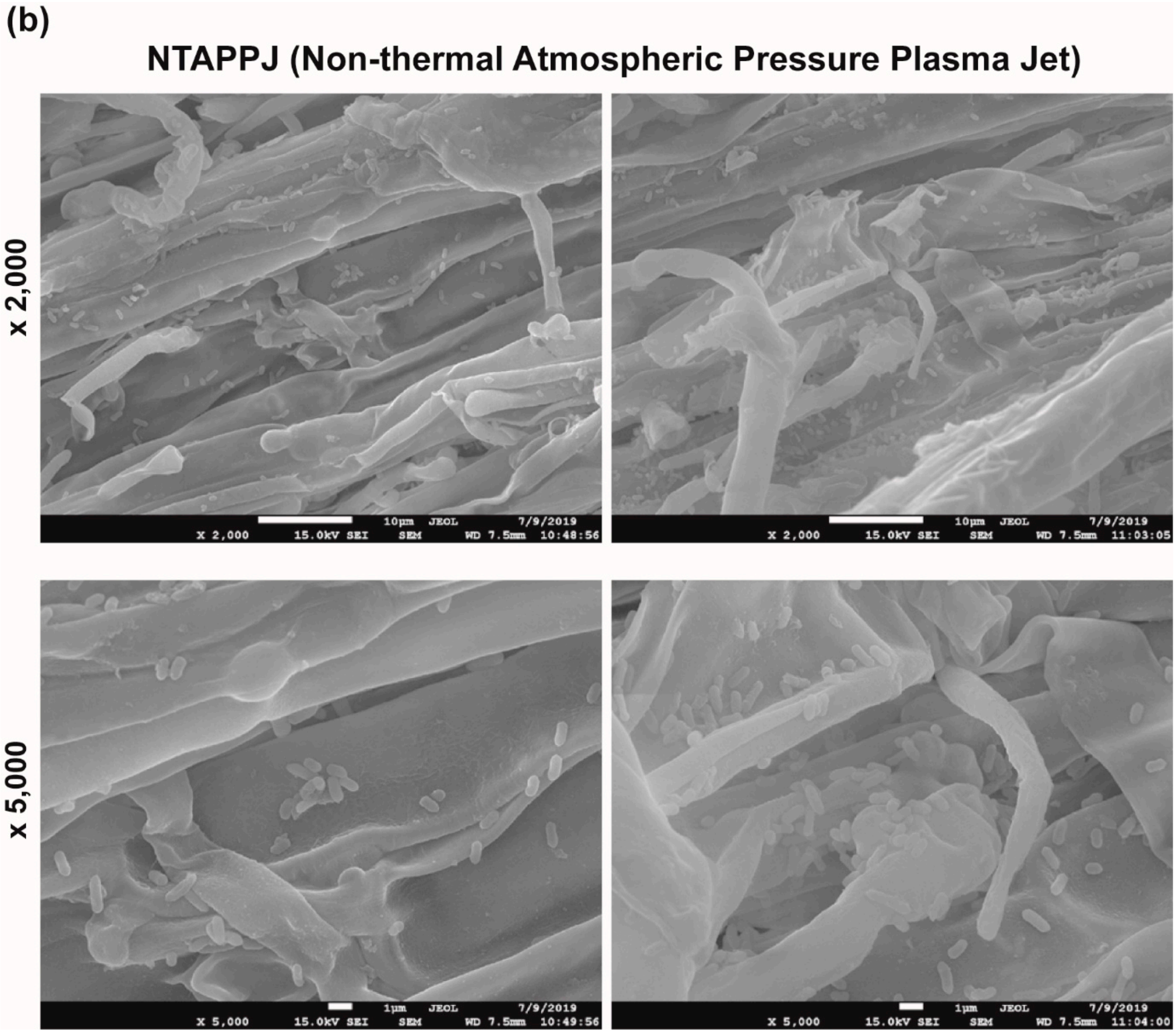
(b)
NTAPPJ (Non-thermal Atmospheric Pressure Plasma Jet)
x 2,000
x 5,000
10μm JEOL 7/9/2019
X 2,000 15.0kV SEI SEM WD 7.5mm 10:48:56
10μm JEOL 7/9/2019
X 2,000 15.0kV SEI SEM WD 7.5mm 11:03:05
1μm JEOL 7/9/2019
X 5,000 15.0kV SEI SEM WD 7.5mm 10:49:56
1μm JEOL 7/9/2019
X 5,000 15.0kV SEI SEM WD 7.5mm 11:04:00

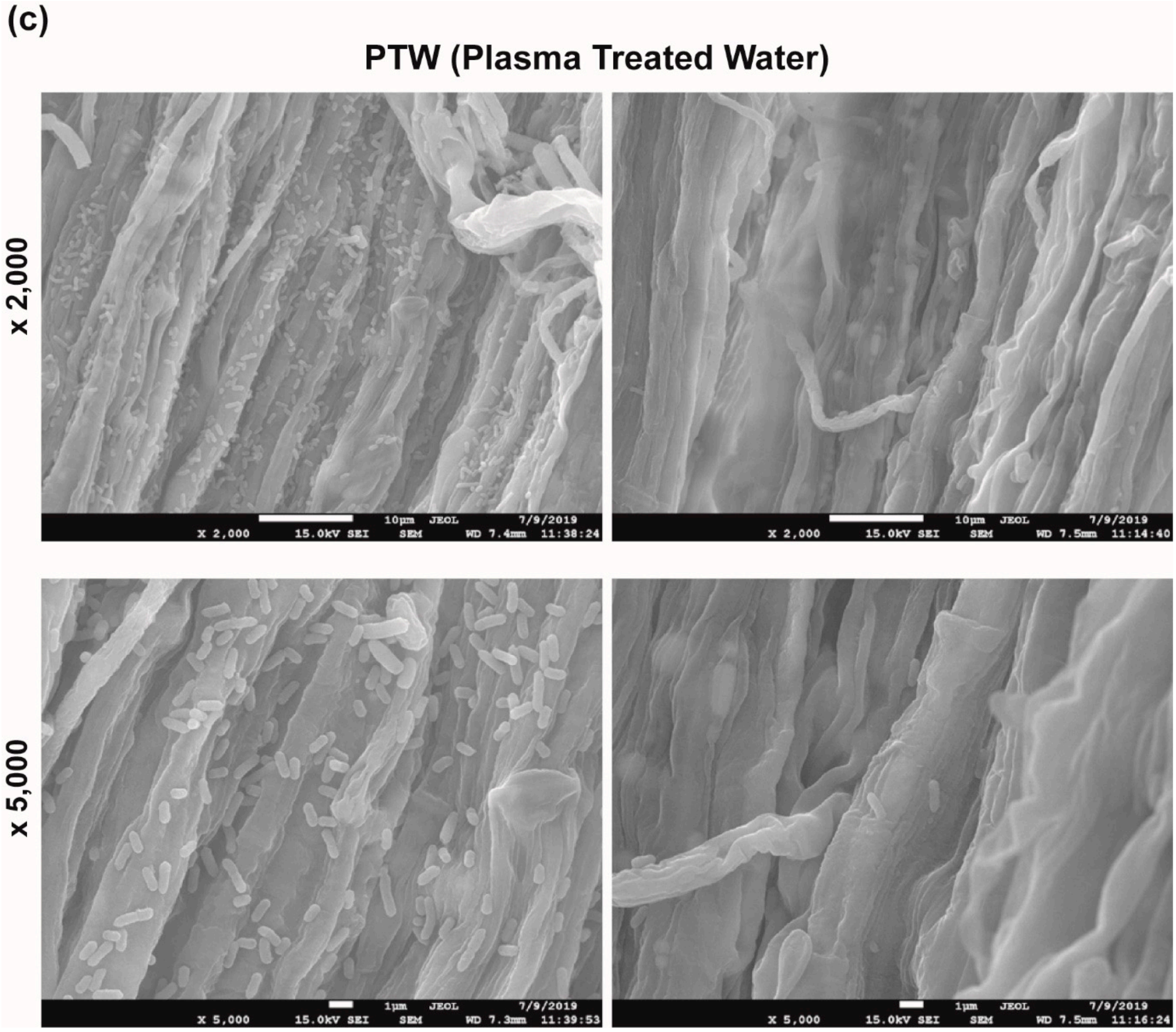

Figure S1. SEM analysis of a mushroom surface after bacteria were inoculated. Mushroom surfaces were treated with none (**a**), NTAPPJ (**b**), and PTW (**c**) for 15 min.

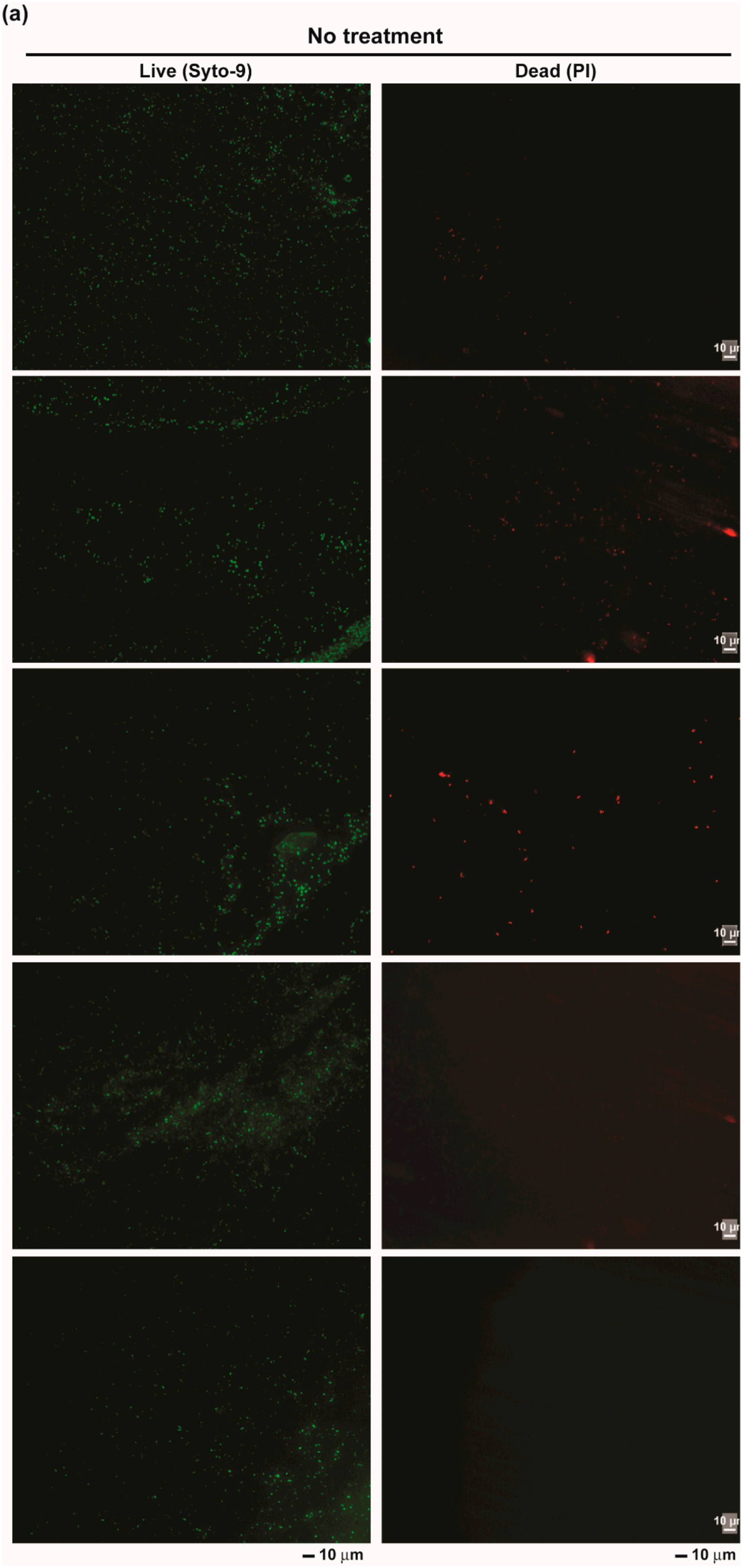
(a)
No treatment
Live (Syto-9)
Dead (PI)
10 μm
10 μm

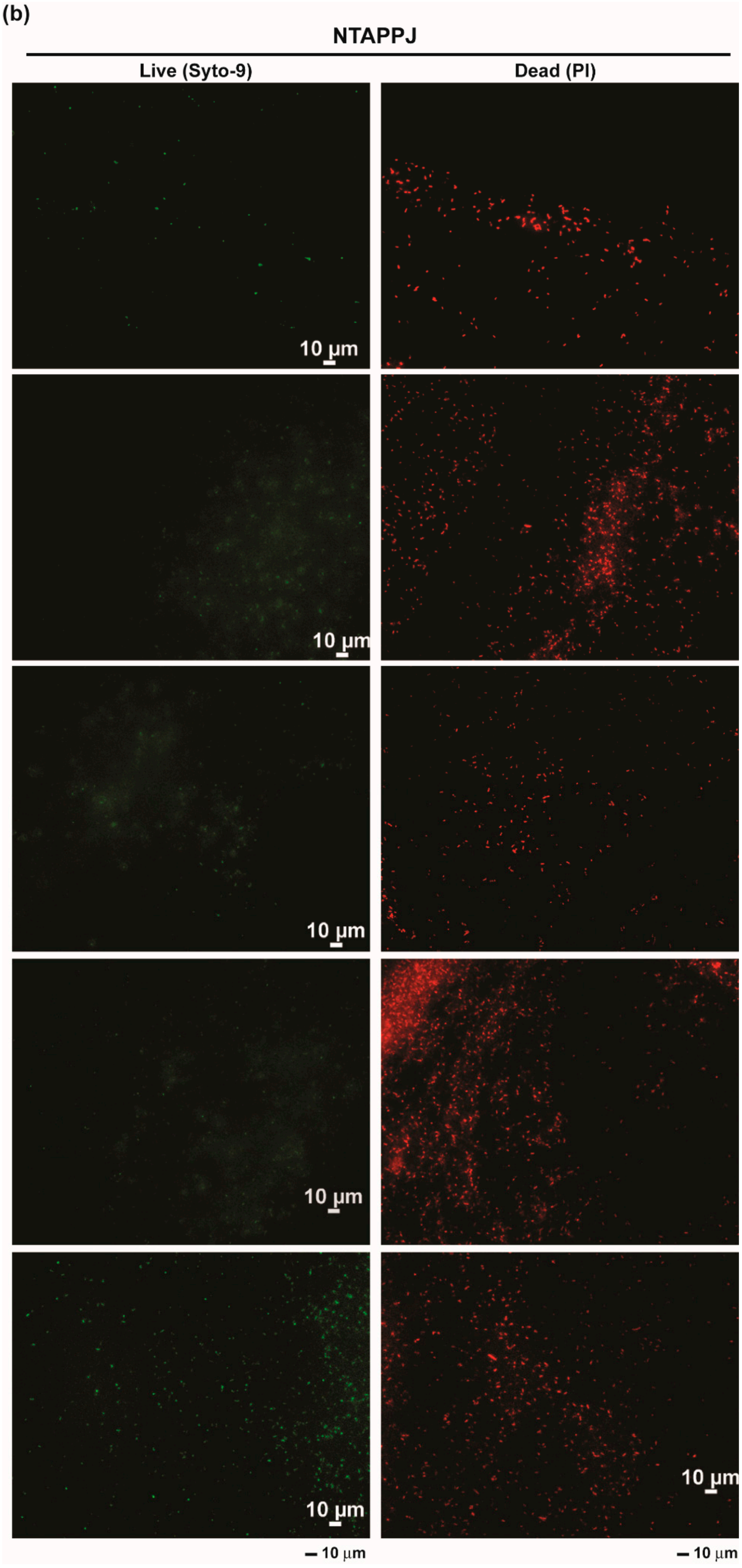
(b)
NTAPPJ
Live (Syto-9)
Dead (PI)
10 µm
10 µm
10 µm
10 µm
10 µm
10 µm
10 µm
10 µm

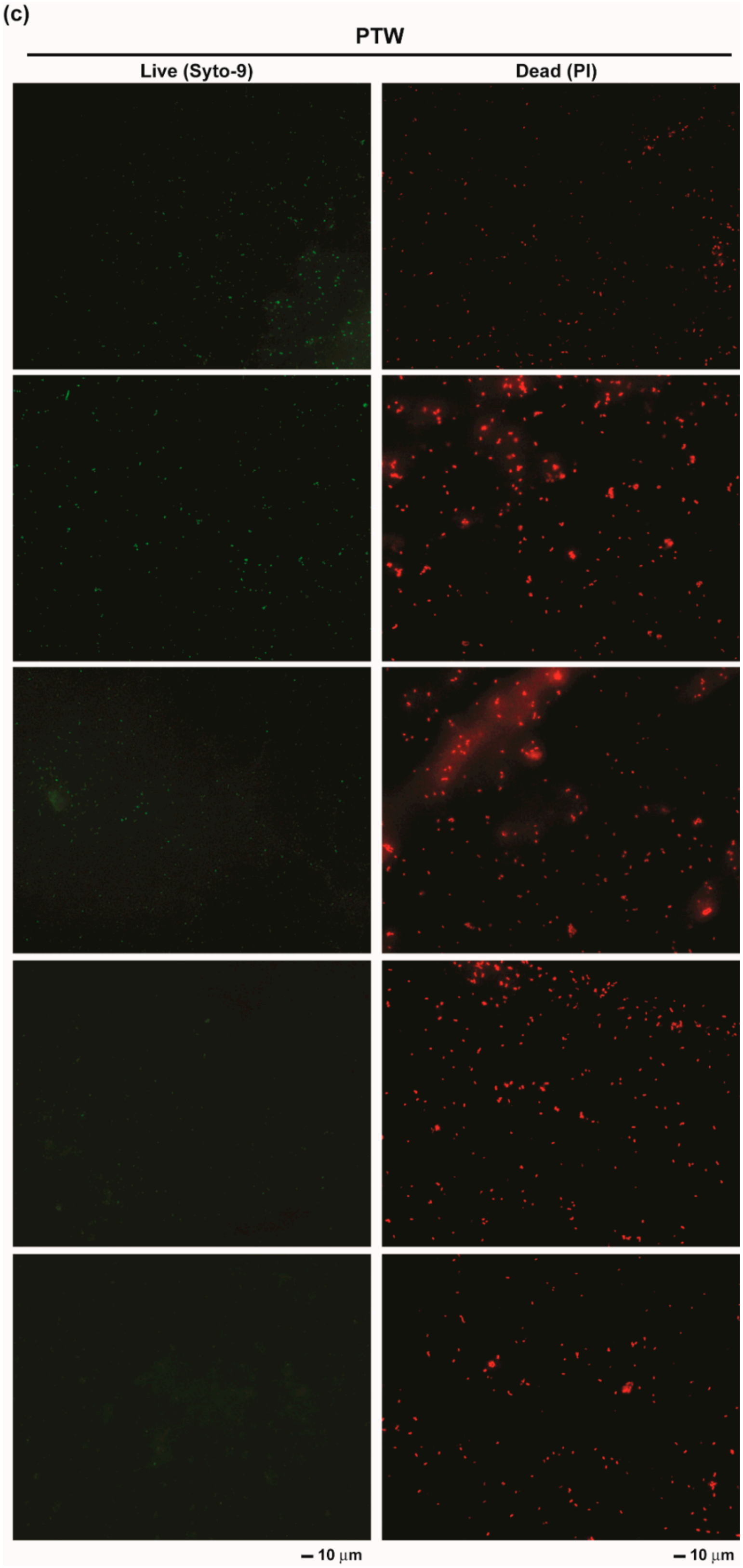

Figure S2. Live (green fluorescence) and dead (red fluorescence) bacteria on mushroom surfaces treated with none (**a**), NTAPPJ (**b**), and PTW (**c**). Mushroom surfaces were treated for 15 min and then inoculated with bacteria.

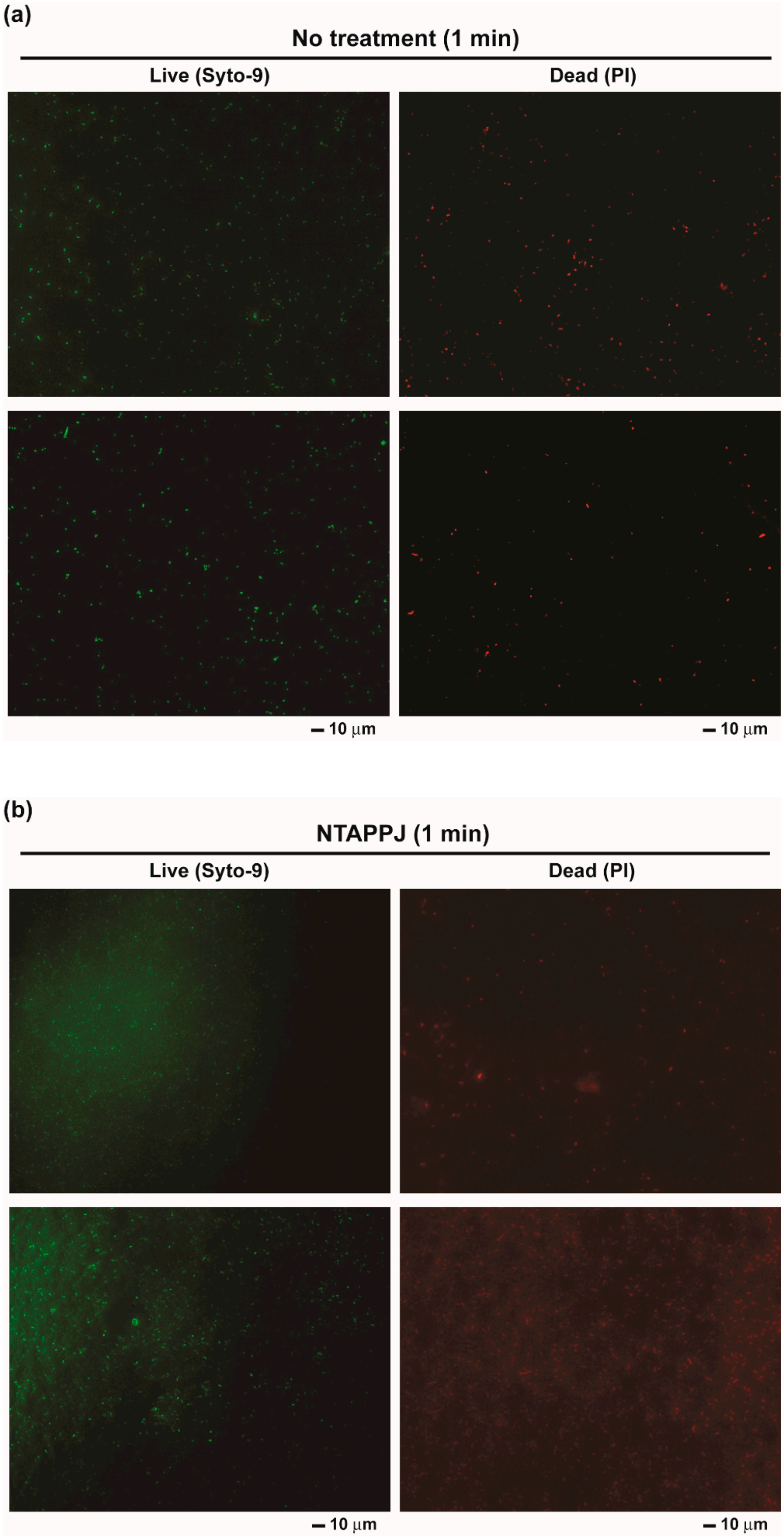
(a)
No treatment (1 min)
Live (Syto-9)
Dead (PI)
10 μm
10 μm
(b)
NTAPPJ (1 min)
Live (Syto-9)
Dead (PI)
10 μm
10 μm

(c)

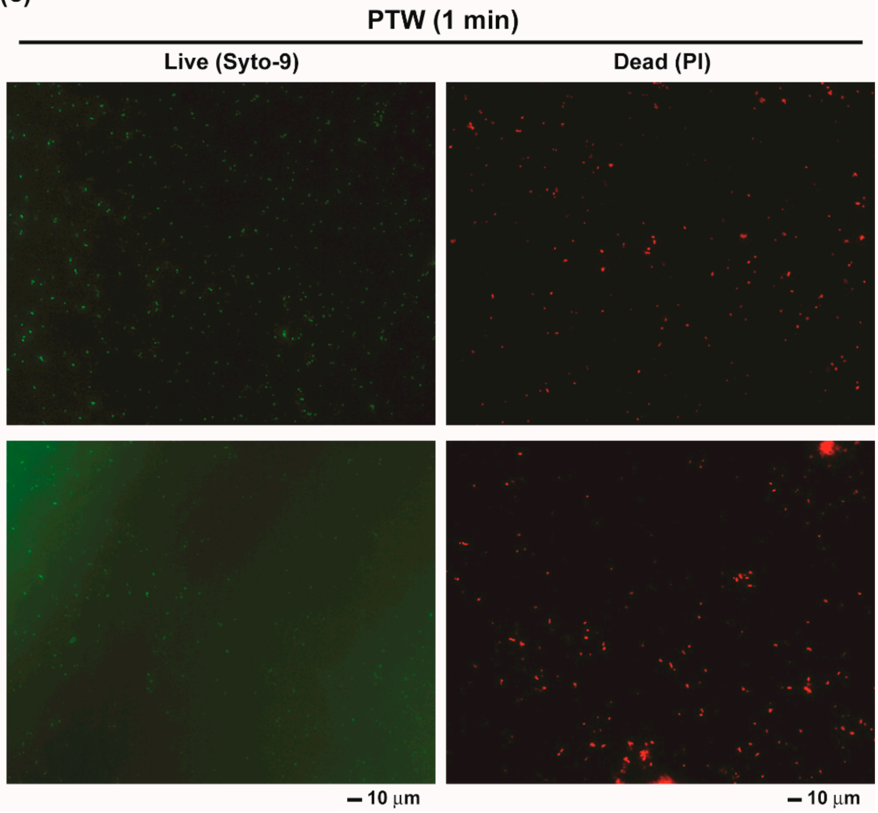
PTW (1 min)
Live (Syto-9)
Dead (PI)
10 μm
10 μm

(d)

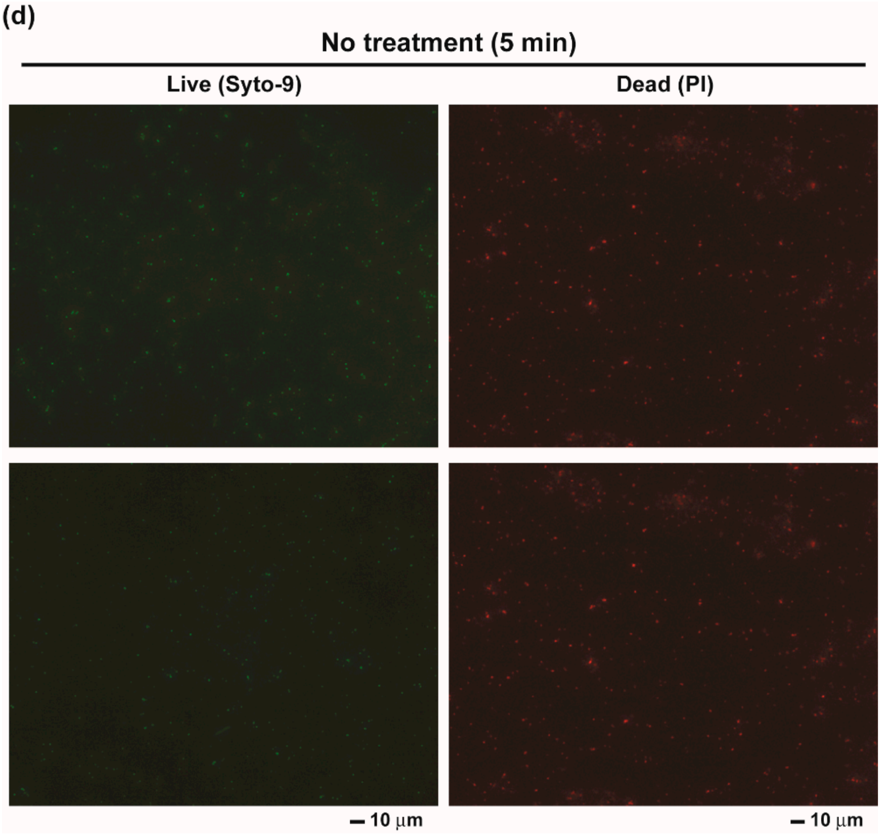
No treatment (5 min)
Live (Syto-9)
Dead (PI)
10 μm
10 μm

(e)

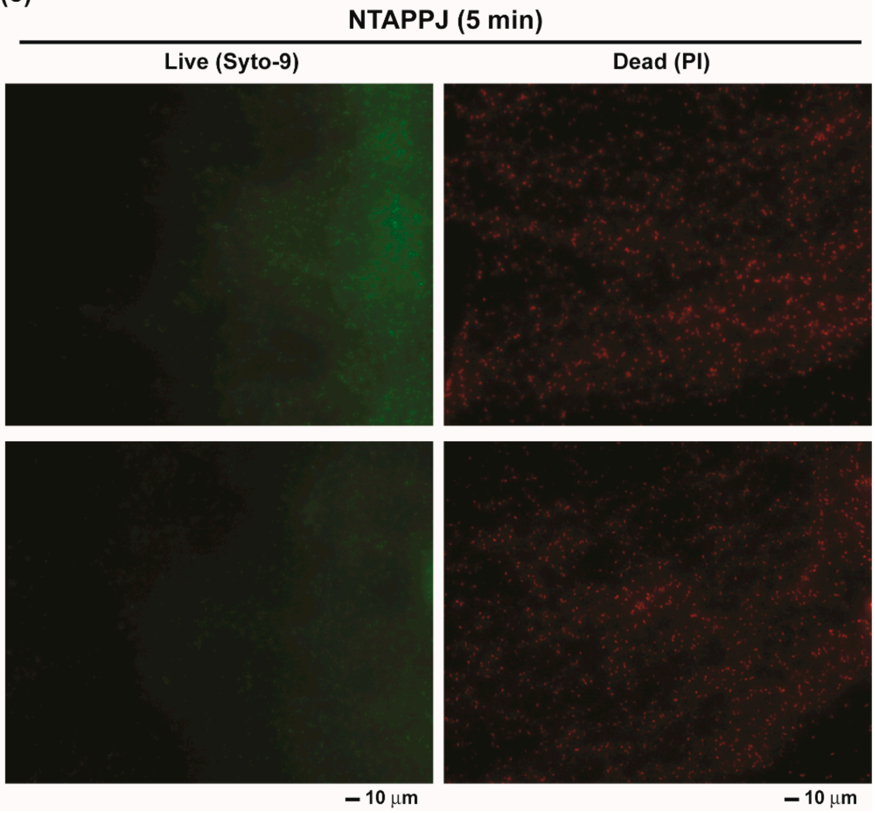

(f)

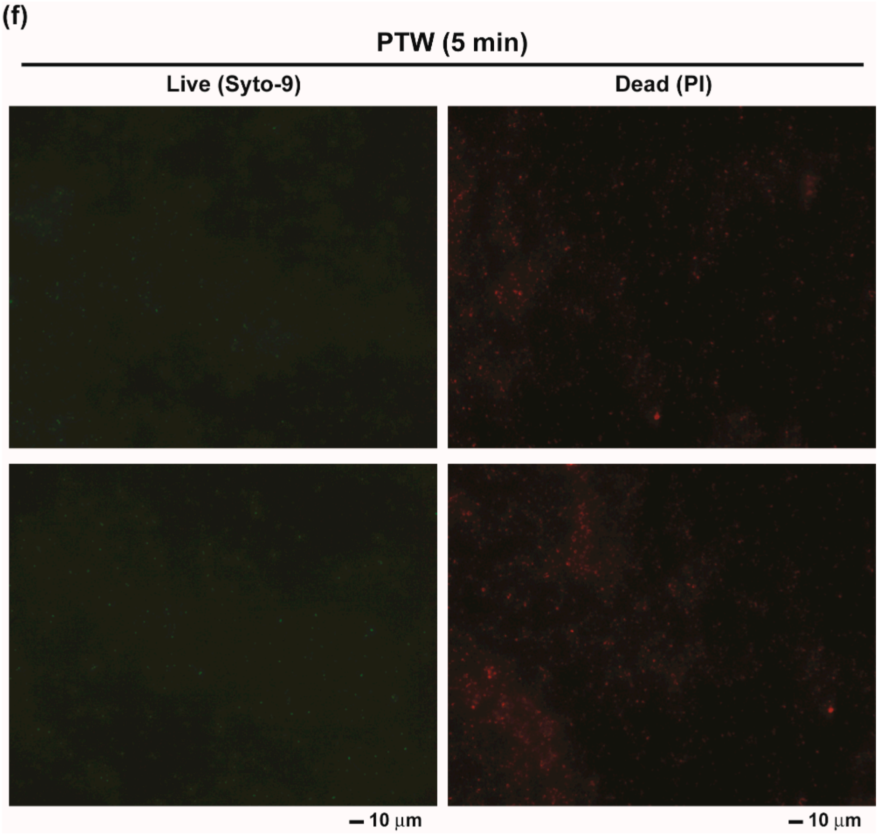

Figure S3. Live and dead bacteria on mushroom surfaces treated with none (**a**,**d**), NTAPPJ (**b**,**e**) and PTW (**c**,**f**) after incubation for 1 min (**a**–**c**) and 5 min (**d**–**f**). Mushroom surface was

treated with NTAPPJ or PTW for 15 min and then inoculated with bacteria. After brief washing and drying to remove non-adherent bacteria, mushroom was incubated for 1 and 5 min.

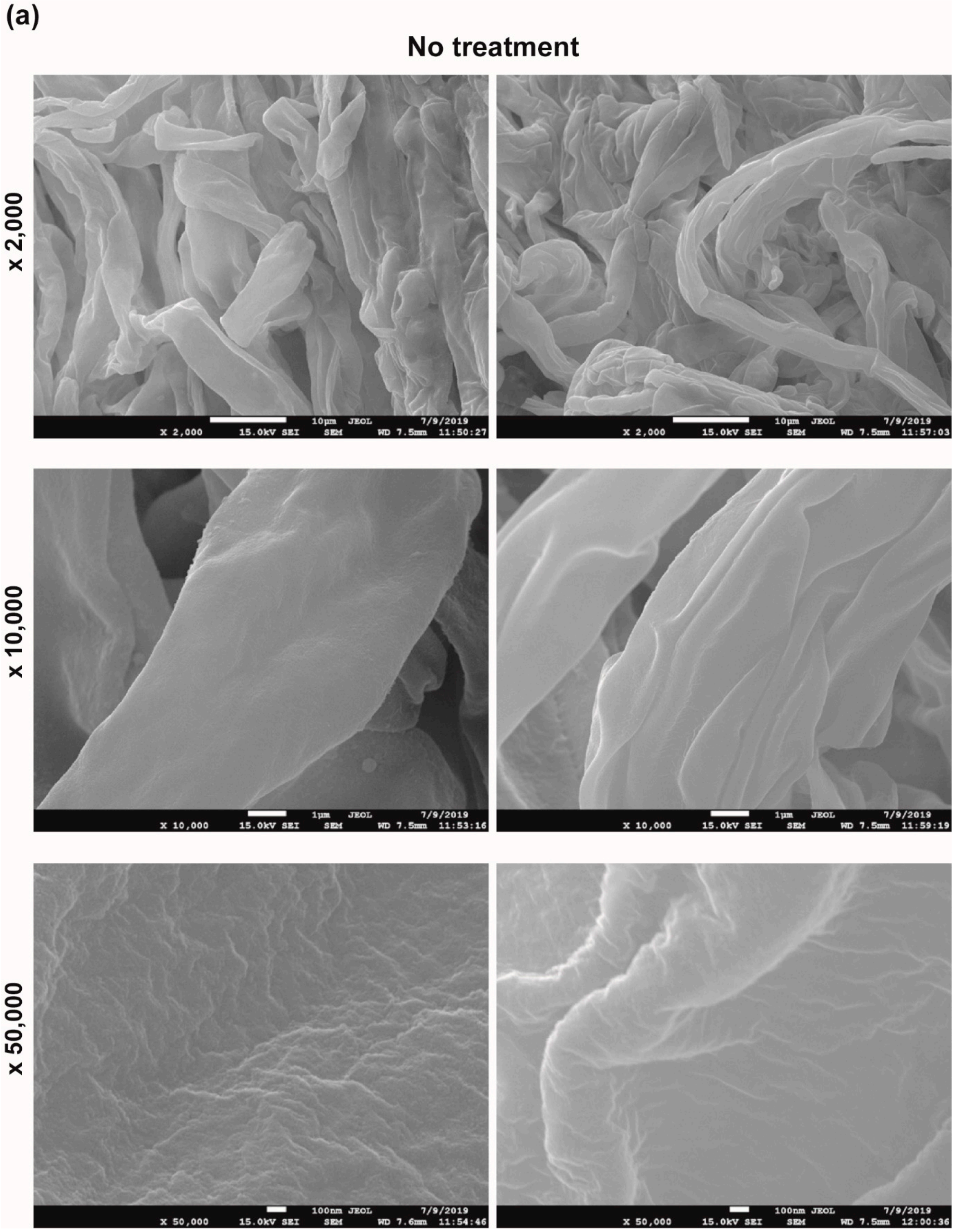

(b)

NTAPPJ (Non-thermal Atmospheric Pressure Plasma Jet)

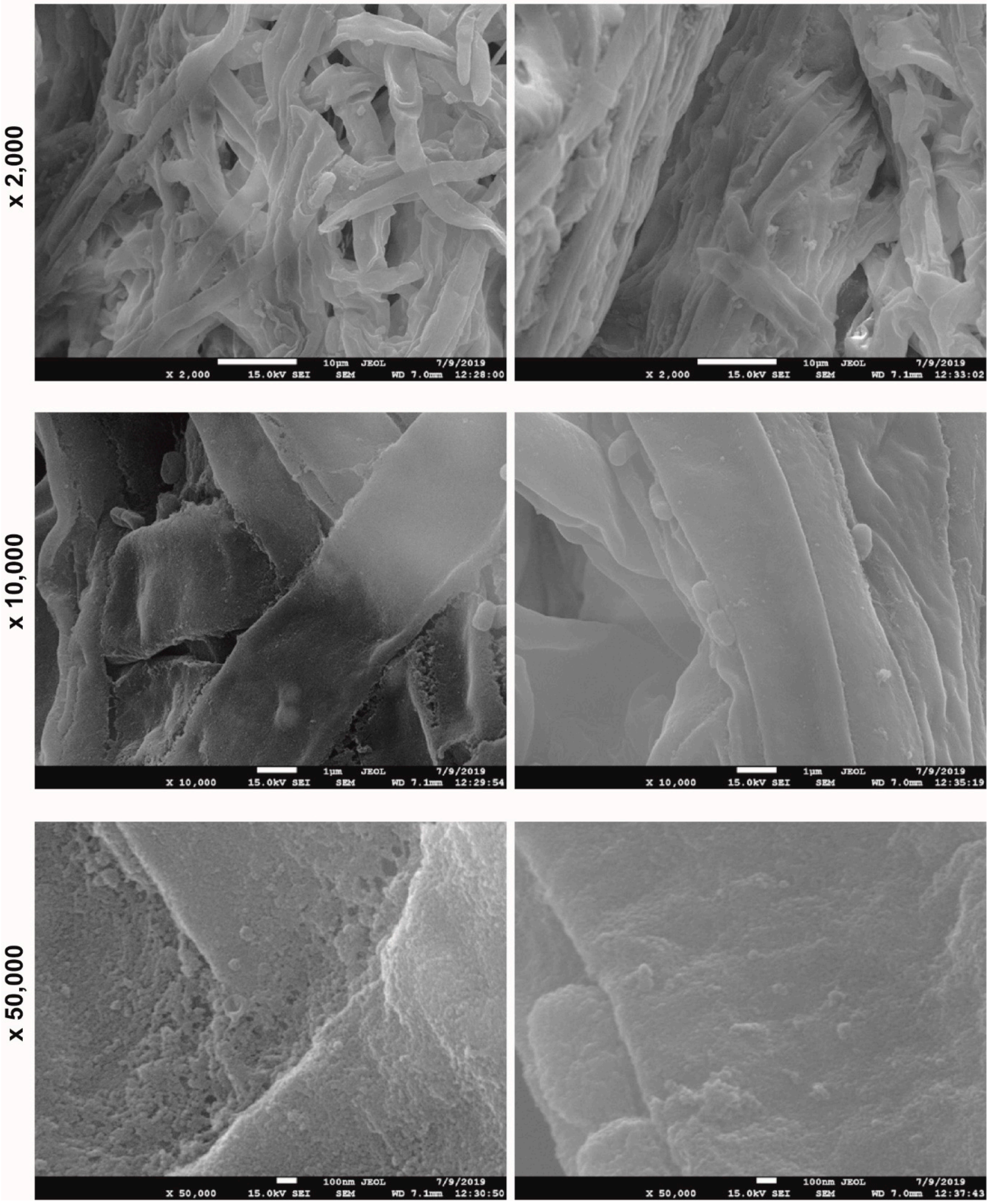

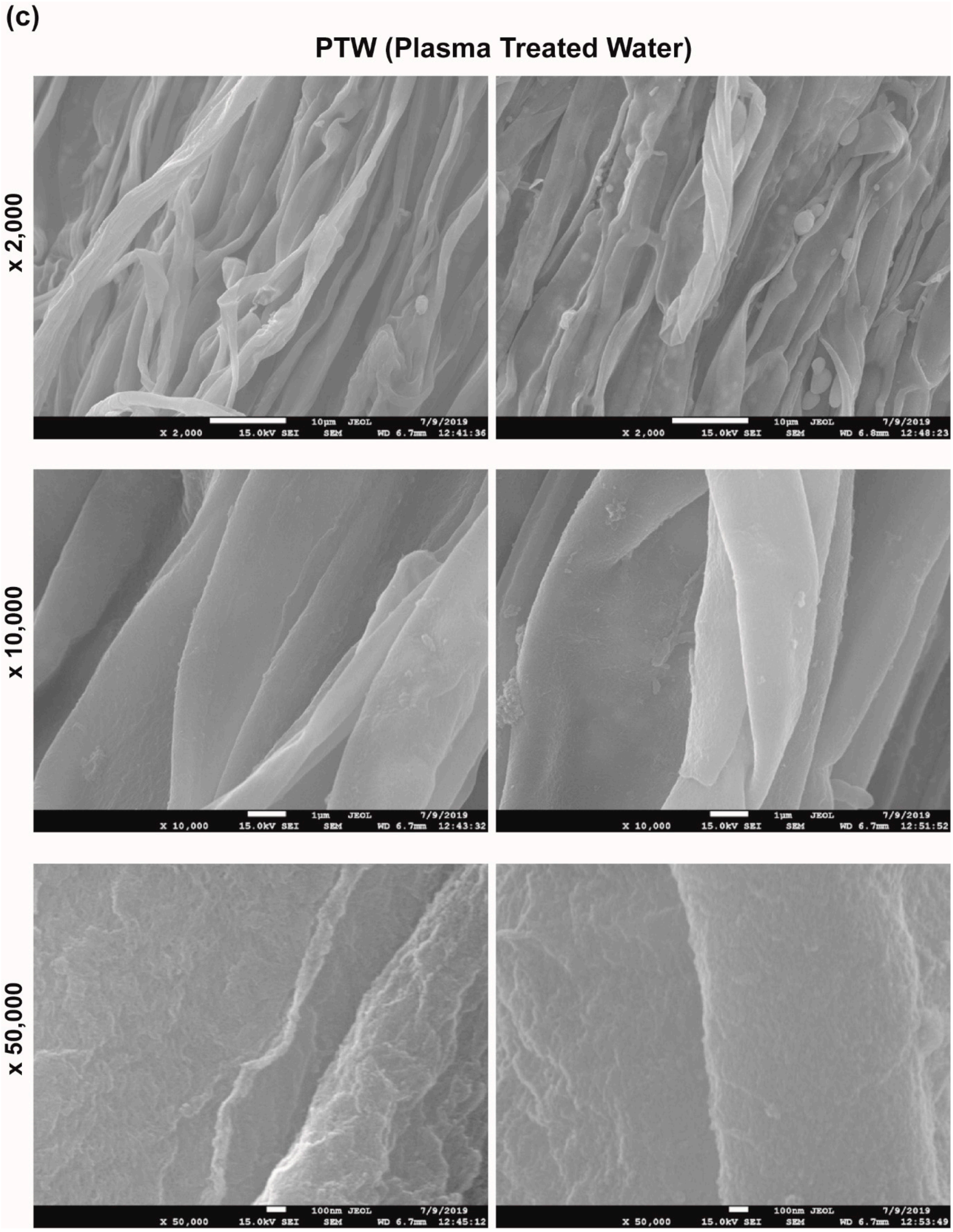

Figure S4. Mushroom surfaces analyzed by SEM after treated with none (**a**), NTAPPJ (**b**), and PTW (**c**) for 15 min.
